# Genetic association of *FKBP5* with trait resilience in Korean male patients with alcohol use disorder

**DOI:** 10.1038/s41598-021-98032-6

**Published:** 2021-09-16

**Authors:** Chun Il Park, Hae Won Kim, Syung Shick Hwang, Jee In Kang, Se Joo Kim

**Affiliations:** 1 Department of Psychiatry, CHA Bundang Medical Center, CHA University, Seongnam, Republic of Korea; 2 Institute of Behavioral Science in Medicine, Yonsei University College of Medicine, Seoul, Republic of Korea; 3 Department of Psychiatry, Yonsei University College of Medicine, 50-1 Yonsei-ro, Seodaemun-gu, Seoul, 03722 Republic of Korea; 4 Department of Medical Education, Yonsei University College of Medicine, Seoul, Republic of Korea; 5 Yonsei Empathy Psychiatric Clinic, Gunpo, Republic of Korea

**Keywords:** Genetics research, Behavioural genetics

## Abstract

The *FKBP5* gene is known to have an important role in alcohol use disorder (AUD) in response to stress and has been reported to affect stress responses by interacting with childhood trauma. This study investigated the effects of the *FKBP5* polymorphism rs1360780 and childhood trauma on trait resilience in male patients with AUD. In addition, allele-specific associations between *FKBP5* DNA methylation and resilience were examined. In total, 297 men with AUD were assessed for alcohol use severity, childhood trauma, resilience, and impulsivity. Genotyping for *FKBP5* rs1360780 and DNA methylation were analyzed. The effects of the rs1360780 single nucleotide polymorphism (SNP) and clinical variables on resilience were tested using linear regression analysis. Possible associations between *FKBP5* DNA methylation and resilience were tested with partial correlation analysis. The rs1360780 risk allele, a low education level, and high impulsivity were associated with diminished resilience, whereas no significant main or interaction effect of childhood trauma with the SNP rs1360780 genotype on resilience was shown. No significant association between *FKBP5* DNA methylation and resilience was found. The present study demonstrated the involvement of the rs1360780 risk allele in trait resilience in men with AUD, suggesting that the genetic vulnerability of *FKBP5* may influence resilience related to AUD.

## Introduction

Alcohol use disorder (AUD) is a complex psychiatric disorder that contributes to the global death and disease burden^1^. Substantial evidence on AUD supports the role of stress in the development of alcohol dependence and the risk of relapse^2^. In particular, biological studies in patients with AUD have shown that the hypothalamic–pituitary–adrenal (HPA) axis and glucocorticoid dysfunction were associated with problematic alcohol use and relapse vulnerability during abstinence^3^. In addition, studies of risk resilience to alcohol addiction indicated that exposure to childhood adversity is linked to the risk and severity of AUD later in life^4–6^, whereas trait resilience, which is the ability to adapt and thrive despite adversity, is protective against AUD^7,8^. Although the underlying molecular mechanisms between stress and AUD-related pathophysiology are unclear, complex interactions between an individual’s genetic predisposition and stress exposure may play a role in the risk-resilience balance of AUD.

FKBP51 is a major regulatory protein of the HPA axis and is known to modulate glucocorticoid receptor (GR) sensitivity by binding to GRs in the cytosol and reducing GR nuclear translocation in response to stressors^9–11^. It is an important molecular target of human stress responses and psychiatric disorders, including AUD^12,13^. Genetic polymorphisms of *FKBP5* have been shown to have functional effects on the HPA axis and *FKBP5* gene expression^14,15^, and *FKBP5* expression levels are considered to be induced in an allele-specific manner and to interact with childhood trauma^14^. Recently, several *FKBP5* gene variants have been reported to be associated with alcohol withdrawal severity, and the effect following chronic alcohol exposure was validated using *FKBP5* -knockout mice^12^. In particular, rs1360780 in *FKBP5*, the most widely studied genetic polymorphism in stress-related psychiatric disorders, was reported to have an interaction effect with childhood trauma on predicting heavy alcohol consumption (a risk factor for AUD) in nonclinical college students^16^.

DNA methylation, an epigenetic mechanism, is known to be modulated by environmental factors and regulates gene expression^17^. A growing body of literature has investigated *FKBP5* methylation as a proxy for the interaction between genes and stressful situations, such as childhood adversity^18^. A study of Holocaust survivors and their offspring suggested that altered *FKBP5* methylation levels at intron 7 were evident in exposed parents and their siblings^19^. In another study of children who were cared for in institutions at an early age^20^, the time spent in institutions was negatively associated with the methylation level of *FKBP5*. Recently, changes in DNA methylation of *FKBP5* associated with adverse childhood events have been observed in various stress-related psychiatric disorders, including post-traumatic stress disorder (PTSD)^21^, depression^22^, psychosis^23^, and borderline personality disorder^24^.

A recent study in a healthy population showed a significant interaction effect between *FKBP5* and childhood trauma on trait resilience, where the rs1360780 risk allele enhanced the negative impact of childhood neglect on resilience in women^25^. Interestingly, rs1360780 was reported to interact with DNA methylation in the *FKBP5* gene and consequently alter stress-dependent gene transcription and dysregulate the neuroendocrine system^14^. These findings suggest that genetic factors and epigenetic regulation of *FKBP5* may play an important role in stress resilience among individuals with AUD.

Here, we investigated the main and interactive effects of rs1360780 and childhood trauma on trait resilience, a protective factor for stress-related psychopathology, in Korean male patients with AUD. In addition, we examined whether allele-specific associations exist between DNA methylation levels at intron 7 of *FKBP5* and the patients’ resilience scores.

## Results

### Demographic and clinical characteristics

The demographic and clinical characteristics of the participants with AUD are presented in Table 1 according to genotype groups of *FKBP5* rs1360780. No significant differences in demographics, clinical characteristics, or *FKBP5* DNA methylation were found between CC homozygous and T-allele carriers of rs1360780. The genotype distributions of rs1360780 were as follows: genotype CC, 172 (57.9%) participants; genotype TC, 105 (35.4%) participants; and genotype TT, 20 (6.7%) participants. The genotype distributions in the present sample with AUD were not significantly different from those in Korean samples of previous studies with major depressive disorder^26^ and PTSD^27^. The number of participants with or without childhood trauma by rs1360780 genotype did not significantly differ.

**Table 1:** Descriptive summary of demographic, clinical, and epigenetic characteristics of patients with AUD based on the genotype groups of *FKBP5* rs1360780.

	*FKBP5* rs1360780^a^		*p*-value^b^
	CC (n = 172)	TC + TT (n = 125)	
Age, years	48.72 ± 7.86	47.77 ± 7.34	0.292
Education, years	13.78 ± 3.87	13.30 ± 3.98	0.289
Duration of AUD, years	17.14 ± 9.92	16.85 ± 9.89	0.805
AUDIT	26.85 ± 7.91	26.89 ± 7.44	0.966
BIS	51.41 ± 11.41	50.25 ± 10.56	0.371
RQT	16.70 ± 22.90	12.34 ± 20.33	0.096
**Childhood trauma (n)**			
Higher	82	66	0.383
Lower	90	59	
***FKBP5* methylation (%)**			
CpG1	96.38 ± 2.69	96.52 ± 2.69	0.643
CpG2	92.80 ± 2.49	92.57 ± 2.29	0.419

*AUD* alcohol use disorder, *AUDIT* the Alcohol Use Disorders Identification Test, *BIS* Barratt Impulsiveness Scale, *RQT* Resilience Quotient Test.

^a^Mean ± standard deviation except for childhood trauma.

^b^Independent *t*-test or chi-squared test.

### Regression model for resilience prediction

The multiple regression model included the rs1360780 genotype (CC vs. T-allele carriers), childhood trauma (higher vs. lower trauma exposure), and their interaction term. In addition, the model included several demographic and clinical variables that showed significant associations with resilience scores from correlation analyses, including duration of AUD, educational status, and trait impulsivity (Supplementary Table S1). The present sample size of 297 was powered to detect small to medium effects (Cohen’s f-square: 0.027) with a power of 0.8, an alpha value of 0.05, and six predictors using G*Power^17^. Stepwise linear regression analysis revealed three significantly associated factors of trait resilience: the *FKBP5* rs1360780 genotype, trait impulsivity, and education level (Table 2). The final regression model with the three factors accounted for 51.1% of the variance in trait resilience. The different genotypes of rs1360780 explained 1.4% of the resilience score variance. With regard to the directions of effects, the rs1360780 genotype showed a negative beta weight, indicating that the minor T allele confers lower resilience. In addition, while high trait impulsivity had a negative effect on trait resilience, a high education level had a positive effect on trait resilience. On the other hand, the stepwise regression analysis revealed no significant main effect of childhood trauma exposures on trait resilience. In addition, no significant interaction effect between the *FKBP5* genotype and childhood trauma was found.

**Table 2:** Stepwise linear regression analysis^a^ for predicting trait resilience in patients with alcohol use disorder.

	B	SE	β	T	*P*
Constant	73.567	5.575		13.196	< 0.001
Impulsivity	− 1.415	0.085	− 0.683	− 16.698	< 0.001
Educational level	1.154	0.239	0.197	4.82	< 0.001
*FKBP5* T-carrier^b^	− 5.438	1.899	− 0.117	− 2.864	0.004

^a^Model summary: R^2^ = 0.511, adjusted R^2^ = 0.506, F = 101.89, *P* < 0.001.

^b^*FKBP5* SNP rs1360780: T allele carrier was coded as 1, CC genotype was coded as 0.

### *FKBP5* genotypes, *FKBP5* methylation, and resilience

The DNA methylation levels at *FKBP5* intron 7 were not significantly different between the genotype groups of *FKBP5* rs1360780 (CC vs. T-allele carriers) (Table 1). In addition, no significant differences in DNA methylation levels at *FKBP5* intron 7 were found between groups with higher and lower childhood trauma (Supplementary Table S2) or between genotypes (the TT/TC vs. CC genotype) of SNP rs1360780 depending on childhood trauma (Supplementary Table S3).

Partial correlation analyses were conducted between *FKBP5* DNA methylation levels and trait resilience depending on the FKBP5 rs1360780 genotype. Since education level and trait impulsivity were significantly associated with resilience scores in the stepwise linear regression analysis, these two variables were adjusted for in the partial correlation analyses. Trait resilience based on the Resilience Quotient Test (RQT) score had no significant association with DNA methylation levels at *FKBP5* cytosine-phosphate-guanine (CpG) 1 and CpG2 sites in either rs1360780 CC homozygous (r = − 0.131 and *p* = 0.088 for CpG1; r = 0.068 and *p* = 0.381 for CpG2) or T-allele carriers (r = − 0.118 and *p* = 0.194 for CpG1; r = − 0.013 and p = 0.888 for CpG2). Partial correlation analysis between the *FKBP5* methylation level and trait resilience showed that the sample sizes of the rs1360780 CC genotype group (n = 172) and T-allele carrier group (n = 125) were sufficient to detect small to medium effects (Pearson’s r = 0.232 and 0.232, respectively) assuming a power of 0.8 and an alpha value of 0.025.

## Discussion

The present study showed that the *FKBP5* rs1360780 risk allele, a low education level, and high impulsivity were associated with diminished resilience in Korean male patients with AUD, whereas no significant main or interaction effect of childhood trauma with the *FKBP5* gene on resilience was shown. No significant association was found between DNA methylation of *FKBP5* intron 7 and resilience scores according to the genotype groups of rs1360780. The present study is the first to demonstrate the involvement of an *FKBP5* genetic variant in stress resilience among patients with AUD.

Alterations in stress responses and GR sensitivity, which are mediated by the HPA axis, are known to play a key role in coping mechanisms^28,29^ and in the development of stress-related psychiatric disorders^30,31^. A key regulator of the HPA axis is FKBP51, which reduces GR affinity for cortisol in response to stress in both the brain and periphery^32^. Increased FKBP51 protein expression and risk alleles of common polymorphisms, such as the rs1360780 T allele in the *FKBP5* gene, have been reported to be associated with higher GR resistance, dysregulation of the HPA axis, and a maladaptive stress response^9,32,33^. In addition, a translational study showed stress-induced changes in neuroendocrine activity and coping in both *FKBP5*-deficient mice and human subjects with different *FKBP5* genotypes, suggesting a role of *FKBP5* in shaping stress-related phenotypes^34^. Given the biological effects of *FKBP5* in stress-related psychopathology and coping, our findings suggest that *FKBP5* may have a crucial role in modulating the risk-resilience balance in the psychophysiology of AUD through neuroendocrine regulation and stress responses.

The direction of the association with trait resilience indicated that AUD patients with the minor T-allele of rs1360780 had lower resilience than those with the homozygous C allele, which is consistent with previous findings. These findings indicate that the C allele may have a protective effect against stress in AUD patients. Indeed, although little evidence regarding AUD is available, many studies support the idea that while the T allele of rs1360780 plays a severe pathological role in other stress-related psychiatric disorders, such as PTSD^21^ and depressive disorder^35^, while the C allele plays a protective (resilient) role. The effect of *FKBP5* genetic variants should be replicated for stress-related endophenotypes in other populations with AUD.

While we identified *FKBP5* genotype-dependent differences in stress resilience, we found no interaction effect between childhood trauma and the rs1360780 genotype or an effect of childhood trauma alone on resilience, which is inconsistent with previous findings. The interaction effect of childhood trauma with common *FKBP5* allelic variations has been reported to influence susceptibility to stress-related psychiatric disorders^36^. In addition, their interaction effect was shown on the cortisol response after acute stress^37^. Despite limited evidence in AUD, a genetic study in nonclinical college students showed no main effect of the rs1360780 genotype but a significant interaction effect of the genotype on the probability of heavy drinking^16^. Another study in a nonclinical sample showed that while the rs1360780 genotype did not show significant main effects on resilience, a significant interaction effect between the genotype and childhood neglect was found on resilience^25^. These inconsistent results might be partially explained by differences in disease status or chronic conditions and alcohol exposure and by gender differences in stress responses. Further studies are needed to establish the complex relationship of childhood trauma and *FKBP5* with resilience factors related to AUD.

On the other hand, we found no significant difference in DNA methylation at *FKBP5* intron 7 according to the genotypes. Similarly, Menke et al. did not find any genotype‐dependent differences in either *FKBP5* mRNA expression or cortisol levels following dexamethasone stimulation in healthy subjects^33^. Contrary to these results, several studies have shown the effect of allele-specific *FKBP5* DNA methylation levels in stress-related conditions^14,22,27^. For instance, the DNA methylation level at *FKBP5* intron 7 has been associated with childhood trauma in an allele-specific manner^14^. Different characteristics of participant samples, such as the chronicity and severity of AUD, may contribute to these inconsistencies in study findings. In addition, possible confounding factors, including interindividual differences in white blood cell counts and cumulative effects of repeated exposure to alcohol, cigarettes^38^, and psychotropics^39^, may explain some of the inconsistent findings. Further investigations are needed to clarify *FKBP5* genotype‐dependent differences in DNA methylation and the role of the epigenetic status of *FKBP5* in resilience in patients with AUD.

Our study had several limitations. First, we did not include healthy controls, as our main purpose was to examine the effects of genetic factors and childhood trauma on the endophenotype of trait resilience among patients with AUD. Further studies including healthy controls would be helpful to explore differential associations of *FKBP5* and childhood trauma in the context of susceptibility to AUD. Second, when considering gender differences in epidemiological^40^ and clinical characteristics of AUD^41^ and sexually dimorphic effects of HPA axis function in response to stress, the present results are not generalizable to women with AUD, as we included only men in our study. Third, we did not select all tagging SNPs covering the entire *FKBP5* gene. Studies with larger sample sizes and a larger number of SNPs are needed to clearly confirm the results. In addition, we adapted only a hypothesis-based candidate gene approach to the *FKBP5* gene. However, because candidate association studies have several limitations, such as a lack of replication^42^, further studies using hypothesis-free genome-wide association studies with a larger sample size are also warranted. Fourth, we did not measure functional levels of the *FKBP5* gene, such as *FKBP5* mRNA expression and cortisol levels, according to genotype groups. Fifth, since we assessed childhood maltreatment, sexual abuse, and parental conflict through retrospective self-reports, the present study is limited by potential recall bias regarding childhood trauma exposure. Sixth, we determined the ethnicity of the participants with self-reports rather than by ancestry-informative markers. Although the Korean people were considered to be homogenous with respect to ethnic variation^43^, possible biases from population stratification remained. Last, our cross-sectional study could not infer how the relationship between rs1360780 and resilience influences the clinical outcomes of AUD. Further study with a longitudinal design investigating AUD progression or relapse is needed.

The present study demonstrated the involvement of the rs1360780 risk allele in stress resilience among male patients with AUD, suggesting that the genetic vulnerability of *FKBP5* may contribute to an unfavorable stress response and resilience related to AUD, possibly via HPA axis dysregulation. Future studies in larger populations with AUD are needed to elucidate the effects of *FKBP5* and the HPA axis and the underlying biological mechanism of resilience and risk in AUD.

## Methods

### Participants

In total, 297 male patients with AUD participated in this study. All participants were inpatients of psychiatric wards for alcohol detoxification and rehabilitation. They were all confirmed for abstinence from alcohol for at least 7 days before participation in this study. All participants were diagnosed with alcohol dependence according to the Diagnostic and Statistical Manual of Mental Disorders, 4th Edition criteria. The exclusion criteria were as follows: (1) a history of major psychiatric disorder(s), including schizophrenia and other mood or anxiety disorders, and (2) other substance dependencies except for alcohol and cigarettes in the previous 6 months. All participants were of Korean ethnicity, which was determined on the basis of the investigators’ observation and self-reporting by the participants. This study was carried out in accordance with the Declaration of Helsinki. The Institutional Review Boards at Severance Hospital, Seoul, Republic of Korea, reviewed and approved this study (No. 4-2011-0398). All participants were given written informed consent before participating in this study.

### Measurements

The severity of AUD was assessed using the Alcohol Use Disorders Identification Test (AUDIT)^44^. Higher AUDIT scores correspond to more hazardous drinking behaviors. In addition, trait impulsivity (an endophenotype of AUD^45^) was measured using the Korean version of the Barratt Impulsiveness Scale (BIS)^46^.

To evaluate childhood trauma, we investigated maltreatment, sexual abuse, and parental conflict during childhood. First, childhood maltreatment and neglect were assessed using the modified Korean version of the Parent–Child Conflict Tactics Scale (mPCCTS), which is based on the original Parent–Child Conflict Tactics Scale^47,48^. The mPCCTS consists of three subfactors: psychological maltreatment, physical maltreatment, and neglect of children before 12 years of age. Second, the “sexual abuse” section of the Childhood Maltreatment Scale was also used^49^. The scale is composed of minor sexual violence, such as verbal abuse, and severe sexual assault, such as oral sex and sexual intercourse, before the age of 18. Third, the modified version of the Conflict Tactics Scale (mCTS) was used for the childhood experience of parental conflict before 12 years of age^50,51^. Because the distribution of childhood trauma scores was skewed to the left, the scores were dichotomized into higher and lower trauma exposure based on the median composite scores on the mPCCTS, mCTS, and sexual abuse scales; childhood trauma was coded as a binary variable in the analyses.

To assess trait resilience, the RQT was used^52^, which is a 56-item questionnaire consisting of seven factors: emotional regulation, impulse control, realistic optimism, causal analysis, empathy, self-efficacy, and reaching out. The total RQT scores were calculated as an index of trait resilience.

All questionnaires used in this study have been previously validated in Korean populations^47,49,50,53–55^.

### DNA extraction and genotyping

The polymorphism rs1360780 of the *FKBP5* gene was genotyped using DNA extracted from peripheral venous blood from the participants. The detailed genotyping method has been described in a previous study^27^. The forward primer sequence was 5′-CCTGAAAAGATTATCTGATGC-3′, and the reverse primer sequence was 5′-GCAAAGTCTCCACTGTTTCT-3′. In our DNA samples, we obtained a 100% genotyping success rate. The genotype frequencies of rs1360780 were in Hardy–Weinberg equilibrium (*p* = 0.47).

### Pyrosequencing procedures

Genomic DNA was isolated from whole blood cells, and bisulfite pyrosequencing was performed for DNA methylation analysis of *FKBP5* intron 7, which has been extensively investigated, and its methylation level at CpG sites was associated with the genotype of rs1360780, childhood trauma, and transcriptional activation of *FKBP5*^14^. We targeted two CpG sites in the CpG-rich region of *FKBP5* intron 7 based on previous Korean studies reporting an association with major depressive disorder^26^ and PTSD^27^, which were located between 35,590,711 and 35,590,741 of chromosome 6 based on genome build 38 of the NCBI human reference assembly. CpG1 (chr6: 35,590,711) and CpG2 (chr6: 35,590,736) corresponded to + 52,080 bp and + 52,105 bp relative to the transcriptional start site of exon 6, respectively. During pyrosequencing, a non-CpG cytosine was analyzed for internal quality control of the completeness of bisulfite treatment. The pyrosequencing procedures were all carried out at the same time by Genomictree (Daejeon, South Korea). Details of the assay procedures have been previously described elsewhere^27^.

### Statistical analyses

All data were analyzed using SPSS 25.0 software for Windows (SPSS Inc., Chicago, IL, USA). Differences in demographic and clinical characteristics between rs1360780 CC or T-allele carriers were examined using chi-squared tests for categorical variables and *t*-tests for continuous variables. Stepwise linear regression analysis was performed to identify the main and interaction effects of the *FKBP5* gene and childhood trauma on trait resilience, with potentially confounding factors. Before constructing the regression model, to determine which variables might best predict trait resilience in AUD patients, we first calculated bivariate correlations. To avoid multicollinearity in the regression analysis, only variables that were not significantly correlated with each other were included as independent variables in the regression analysis. Potential associated factors, including the *FKBP5* rs1360780 polymorphism, childhood trauma, the interaction between genotype and trauma (gene * trauma), and other demographic variables or clinical characteristics related to AUD, were considered independent variables in the regression models. Additionally, to examine allele-specific associations between *FKBP5* DNA methylation levels and trait resilience, partial correlation analysis was conducted according to the genotype groups of *FKBP5* rs1360780.

## Supplementary Information

Supplementary Information.
