# Supplementary material for: Genetic association of FKBP5 with trait resilience in Korean male patients with alcohol use disorder
Source: Sci Rep. 2021 Sep 16;11:18454. doi: 10.1038/s41598-021-98032-6 (PMC8445975; doi:10.1038/s41598-021-98032-6)
Supplement: Supplementary file 1 — Supplementary Information. [file 41598_2021_98032_MOESM1_ESM.docx]

**Supplementary Information**

| **Supplementary Table S1.** Pearson correlation analysis between trait resilience and demographic/clinical characteristics (n=297) | |
| --- | --- |
| Variables | Trait resilience (RQT)^a^ |
| Age | -0.73 |
| Education | 0.195^*^ |
| Duration of AUD | -0.158^*^ |
| Trait impulsivity (BIS) | -0.675^**^ |
| RQT, Resilience Quotient Test; AUD, Alcohol use disorder; BIS, Barratt Impulsiveness Scale | |
| ^a^Bivariate Pearson correlation | |
| ^*^p<0.01, ^**^p<0.001 | |

| **Supplementary Table S2.** Association between *FKBP5* DNA methylation and childhood trauma | | | |
| --- | --- | --- | --- |
| *FKBP5* methylation (%) | ^a^Childhood trauma | | ^b^*P* value |
|  | Higher (n=148) | Lower (n=149) |  |
| CpG1 | 96.48 ± 2.82 | 96.39 ± 2.55 | 0.77 |
| CpG2 | 92.62 ± 2.52 | 92.79 ± 2.30 | 0.54 |
| ^a^ mean ± standard deviation | | | |
| ^b^ independent *t*-test | | | |

**Supplementary Table S3.** Association between *FKBP5* DNA methylation and rs1360780 genotypes according to childhood trauma

| *FKBP5* methylation (%) | | ^a^Higher childhood trauma | | | ^b^*P* value | | ^a^Lower childhood trauma | | | ^b^*P* value |
| --- | --- | --- | --- | --- | --- | --- | --- | --- | --- | --- |
|  |  | TT/TC (n=66) | CC (n=82) | |  |  | TT/TC (n=59) | CC (n=90) | |  |
| CpG1 | 96.79 ± 2.81 | | | 96.24 ± 2.82 | | 0.24 | 96.23 ± 2.55 | 96.50 ± 2.57 | 0.521 | |
| CpG2 | 92.61 ± 2.22 | | | 92.62 ± 2.75 | | 0.964 | 92.53 ± 2.39 | 92.96 ± 2.24 | 0.267 | |

^a^ mean ± standard deviation

^b^ independent *t*-test
